# Hot-Melt-Extruded Active Films Prepared from EVOH/Trans-Cinnamaldehyde Blends Intended for Food Packaging Applications

**DOI:** 10.3390/foods10071591

**Published:** 2021-07-08

**Authors:** Alejandro Aragón-Gutiérrez, Raquel Heras-Mozos, Miriam Gallur, Daniel López, Rafael Gavara, Pilar Hernández-Muñoz

**Affiliations:** 1 Grupo de Tecnología de Envases y Embalajes, Instituto Tecnológico del Embalaje, Transporte y Logística, ITENE, Unidad Asociada al CSIC, calle de Albert Einstein 1, 46980 Valencia, Spain; miriam.gallur@itene.com; 2 Instituto de Agroquímica y Tecnología de Alimentos, IATA-CSIC, calle del Catedrático Agustín Escardino Benlloch 7, 46980 Valencia, Spain; r.heras@iata.csic.es (R.H.-M.); rgavara@iata.csic.es (R.G.); 3 Instituto de Ciencia y Tecnología de Polímeros, ICTP-CSIC, calle Juan de la Cierva 3, 28006 Madrid, Spain; daniel.l.g@csic.es

**Keywords:** cinnamaldehyde, ethylene vinyl alcohol copolymer, active packaging, melt-extrusion, film properties, antioxidant and antimicrobial packaging

## Abstract

In this work, novel active films based on ethylene vinyl alcohol copolymer (EVOH) and cinnamaldehyde (CIN) were successfully obtained employing a hybrid technique consisting of a two-step protocol involving the preparation of a polymeric EVOH-CIN masterbatch by solvent-casting for its further utilization in the preparation of bioactive EVOH-based films by melt extrusion processing. The influence of CIN over the EVOH matrix was studied in terms of optical, morphological, thermal, and mechanical properties. Optically transparent films were obtained and the incorporation of cinnamaldehyde resulted in yellow-colored films, producing a blocking effect in the UV region. A decrease in the glass transition temperature was observed in the formulations containing cinnamaldehyde, indicating a plasticizing effect. This phenomenon was confirmed by an increase in the elongation at break values of the extruded films. Results from thermogravimetric analysis determined a slight decrease in the thermal stability of EVOH provoked by the vaporization of the bioactive compound. Bioactive properties of the films were also studied; the presence of residual cinnamaldehyde in EVOH after being subjected to an extrusion process conferred some radical scavenging activity determined by the DPPH assay whereas films were able to exert antifungal activity in vapor phase against *Penicillium expansum*. Therefore, the present work shows the potential of the hybrid technique employed in this study for the preparation of bioactive films by a ready industrial process technology for food packaging applications.

## 1. Introduction

Food waste is a major concern for global food security and good environmental politics, directly related to environmental (e.g., energy, climate change, availability of resources), economic (e.g., resource efficiency, costs variability, rising prices, consumption, waste management, commodity markets) and social (e.g., health, equality) impacts [1]. Several works report that between one third and one half of the total food production is not consumed, with negative effects on the entire food supply chain including households [2,3]. As an example, according to the estimations of the United Nations Food and Agriculture Organization (FAO), the losses and spoilage of fruits and vegetables can be as high as 60%, being the highest of all types of foods [4]. Consequently, there is currently a great need to reduce and minimize food losses to make the transition to a resource efficient world. In this line, active packaging systems emerge as a promising solution to improve microbiological food safety and quality and increase the shelf-life of packaged foods [5]. In this regard, the main benefit of antimicrobial active packaging over the direct incorporation of food additives is that the antimicrobial compound is progressively released on the food surface during storage and distribution, causing an inhibitory or lethal effect against pathogen and spoilage microorganisms that affect food products [6,7,8]. From the wide variety of microorganisms with relevance in food packaging applications, fungal pathogens are one of the main causes of food waste and spoilage. Under favorable conditions, fungal spores develop and disperse the fungi, colonizing the produce surface and causing deterioration of the sensory characteristics of the food produce, discoloration, decomposition, and generation of mycotoxins, that may cause health risk. One of the most common fungal pathogens implicated in the deterioration of food produce, mainly of fruits and vegetables, is *Penicillium expansum* [9]. This species is a widespread fungal pathogen in many foods, causing significant economic losses and also health problems associated with the production of toxic secondary metabolites [10,11].

Current trends in active packaging systems restrict the use of synthetic additives in favor of active ingredients extracted from natural bioresources such as essential oils or polyphenol-rich extracts [12,13,14,15]. From the extensive list of essential oils and natural extracts from plants that have demonstrated an inhibitory activity, in this work cinnamaldehyde (CIN) was used as bioactive substance to prepare active packaging films with antifungal activity against *Penicillium expansum*. Cinnamaldehyde is an aromatic α,β-unsaturated aldehyde, and the main ingredient in cinnamon bark and leaf essential oil and has been widely studied as a great antimicrobial compound against a wide range of microorganisms such as bacteria, yeast, and filamentous fungi [8,16,17,18]. CIN presents a melting temperature of −7.5 °C and one of the main advantages of using CIN for the elaboration of antimicrobial films is that the direct contact between the active material and the product is not necessary since the antimicrobial activity of CIN can be exerted in vapor phase. Moreover, CIN is classified as generally recognized as safe (GRAS) by the United States Food and Drug Administration (FDA) [19]. Several works report the incorporation of CIN into different polymeric matrices to prepare active materials intended for food packaging applications. Villegas and co-workers developed bionanocomposite films based on poly (lactic acid) (PLA) reinforced with a nanoclay and were further impregnated with cinnamaldehyde, incorporated through supercritical impregnation employing carbon dioxide [20]. Balaguer et al. incorporated by solvent-casting CIN into gliadin films [21]. Chen et al. reported the encapsulation of trans-cinnamaldehyde into β-cyclodextrin inclusion complex for the preparation of active packaging film incorporating bioactive encapsulated ingredients [22]. Higueras and co-workers immobilized CIN on chitosan films via Schiff base formation, studying their application in active food packaging [23]. Lopes et al. produced films of a cellulose-derivative polymer by solvent casting method incorporating CIN [24]. Most of the cited authors employed the solution casting method to develop polymer based active materials incorporated with CIN. However, this technique is unsuitable for scaling up in an industrial environment, being relegated to be applied for the coating of other surfaces. The main problem of using conventional technologies such as melt extrusion technology is that the process takes places at temperatures above the melting temperature of the polymer matrixes, usually above 170 °C, which may cause the degradation and losses through volatilization of the active compounds [5,25,26]. This work describes an innovative approach for the development of antimicrobial films consisting of the preparation of a polymeric masterbatch containing CIN as bioactive compound, for its further utilization in conventional melt-extrusion processing, thus, minimizing volatile losses due to direct incorporation into the extruder. For that aim, ethylene vinyl alcohol (EVOH) copolymer was selected as polymer matrix. EVOH copolymer is an extensively employed packaging material in the food sector and is considered as one of the most suitable for preparing active materials because of its physicochemical characteristics. It consists of two segment chains: the olefinic, which shows a hydrophobic nature and the vinyl alcohol component, which has a hydrophilic character attributed to the hydroxyl substituent in the vinyl alcohol component [27]. EVOH copolymer has a good chemical resistance, high transparency, and great gas barrier properties to hydrocarbons, especially when a high content of vinyl alcohol is considered [28]. The high susceptibility of EVOH to water, caused by its hydrophilic nature, results in severe plasticization of the copolymer chain when it is in contact with food products with high water activity. This phenomenon can be utilized as an activation mechanism to promote the release or migration of active agents previously added to the EVOH copolymer to the food media [29]. Therefore, the objective of this study is to prepare a polymeric-based masterbatch incorporated with 15 wt.% of CIN for its further utilization in the preparation of bioactive EVOH-based films by melt extrusion processing. The antioxidant and antimicrobial activity of the developed films were evaluated in order to corroborate that CIN was not lost during the extrusion process. Additionally, the effects of CIN on the physicochemical, optical, thermal, and mechanical properties of EVOH-based films were also studied.

## 2. Materials and Methods

### 2.1. Materials

The ethylene vinyl alcohol (EVOH) copolymer utilized in this study was composed of 44% ethylene molar content and was kindly provided by The Nippon Synthetic Chemical Company (Osaka, Japan). Trans-cinnamaldehyde (CIN) and 2,2-diphenyl-1-pricylhydrazyl 95% free radical were purchased from Sigma (Madrid, Spain). 1-propanol and methanol were purchased from VWR International Eurolab (Llinars del Vallès, Barcelona, Spain). Water was obtained from an ultrapure water system Barnstead GenPure Pro (Thermo Fischer Scientific, Waltham, MA, USA).

For the microbiological assays, potato dextrose agar (PDA) was purchased from Scharlau (Scharlab S.L., Barcelona, Spain). The fungal strain *Penicillium expansum* (CECT 2278) was supplied by Spanish Type Culture Collection (CECT) and was maintained in sterile glycerol at −80 °C until its use.

### 2.2. Preparation of the EVOH-CIN Masterbatch

EVOH containing 15 wt.% of CIN was prepared by a solution-extension-evaporation process. In brief, EVOH was dissolved in a 1:1 (*v*:*v*) 1-propanol:Milli Q water solution at 75 °C being stirred for 3 h. Once the EVOH copolymer was completely dissolved, the temperature was lowered to 45 °C and CIN was incorporated to the polymer solution in a proportion of 15 wt.% with respect to the polymer content and stirred for 60 min. Casting was performed using a wire-wound bar coater onto a glass plate. Finally, the obtained material was dried employing a heating tunnel (60 °C) with ventilation for three minutes, and then placing it in a chamber at 40 °C and 14% relative humidity (RH) for 24 h. The developed masterbatch (EVOH-CIN15) was mechanically grinded using a cutting mill SM 2000 (Retsch GmbH, Haan, Germany) to obtain the final material in powder form. The powder was then kept in sealed aluminum foil until its use in the extrusion process.

### 2.3. Characterization of the EVOH-CIN15 Masterbatch

The successful preparation of the masterbatch consisting of EVOH and 15 wt.% of cinnamaldehyde was qualitative evaluated by Fourier Transform Infrared Spectroscopy (FTIR) with a TENSOR 27 Spectrophotometer (Bruker, Massachusetts, USA) coupled to a reflection diamond ATR sampling accessory. Single spectra were recorded in the wavenumber range from 4000 to 650 cm^−1^ at room temperature, averaging 32 scans at a resolution of 4 cm^−1^. Thermogravimetric analysis (TGA) was employed to estimate the final concentration of cinnamaldehyde incorporated in the polymeric masterbatch. For that purpose, a TGA Q-500 thermogravimetric model (TA Instruments, New Castle, DE, USA) was used at a heating rate of 10 °C min^−1^ under a nitrogen flow rate of 50 mL min^−1^.

### 2.4. Film Formation

EVOH-cinnamaldehyde formulations were prepared by extrusion in a XPlore MC15 micro extruder (15 cm^3^ Xplore Instruments BV, Sittard, The Netherlands) being the operating temperatures 175–180 and 185 °C. After a previous drying at 90 °C of EVOH pellets for 4 h, the components were extruded under inert atmosphere by introducing nitrogen into the barrel of the micro extruder, for 3 min, and 70 rpm rotating screw speed. EVOH-CIN15 masterbatch in powder form was blended with pristine EVOH pellets in different proportions to obtain final weight ratios of cinnamaldehyde with respect to EVOH copolymer of 1, 3 and 5 wt.%. The final formulations were immediately injection molded with a 12 cm^3^ micro injector IM12 (Xplore Instruments BV, Sittard, The Netherlands) to obtain dog bone specimens for tensile tests. Films were also obtained thanks to a home-made roll and used for the rest of the characterization tests.

### 2.5. Quantification of Cinnamaldehyde

The content of cinnamaldehyde remaining in both the EVOH-CIN masterbatch and in the bioactive EVOH-CIN films after the extrusion process was quantitatively determined by solid–liquid extraction followed by liquid chromatography mass spectrometry (LC/MS) analysis, as described elsewhere [5]. A calibration curve of cinnamaldehyde was created by injecting known concentrations of the agent into the LC/MS equipment.

### 2.6. Structural and Morphological Properties of Melt Processed EVOH-CIN Films

#### 2.6.1. ATR-FTIR Analysis

FTIR spectra of the developed films were recorded employing the method described in Section 2.3.

#### 2.6.2. Morphological Analysis

Scanning electron microscopy (SEM) was used to study the microstructure of the films along their thickness by observation of their cryofracture surface. Micrographs of the cross sections of the samples were recorded employing a Hitachi S-4800 SEM equipment (Hitachi Ltd., Tokyo, Japan), using an acceleration voltage of 10 kV and a working distance of 8 mm. Films were placed on an aluminum sample holder and were sputtered with a Pd/Au layer to increase the electrical conductivity of the samples.

### 2.7. Thermal Characterization

#### 2.7.1. Differential Scanning Calorimetry (DSC)

A DSC Q-2000 analyzer from TA Instruments (New Castle, DE, USA) was used to evaluate the thermal transitions i.e., glass transition (T_g_), melting (T_m_), and crystallization temperatures (T_c_) as well as their corresponding enthalpies of the developed films. The measurements were performed following the procedure described elsewhere [5]. T_g_ values were determined at the midpoint of heat capacity changes. The melting temperature was obtained from the first and second heating cycles and the degree of crystallinity was determined as described earlier [30], using 217.8 J·g^−1^ as the melting heat associated to a pure crystalline EVOH.

#### 2.7.2. Thermogravimetric Analysis (TGA)

Thermogravimetric analyses of the film samples were performed employing a TGA Q-500 thermogravimetric equipment (TA Instruments, New Castle, DE, USA) as described in Section 2.3. The initial degradation temperature (T_5%_) was determined at 5% weight loss while the temperatures at the maximum degradation rate (T_max_) were calculated from the derivative of the TGA curves.

### 2.8. Functional Properties of the Films

#### 2.8.1. Film Thickness, Transparency and Color Characterization

The color properties of the films were measured in the CIELab space using a KONICA CM-2500d (Konica Minolta Sensing Americas, Inc., Ramsey, NJ, USA), previously calibrated using a white and black standard tile. Optical properties (*L*, lightness; +*a* red, −*a* green; +*b* yellow, −*b* blue), were expressed as average and standard deviation of at least five readings from different parts of the tested film. The total color differences (Δ*E*) induced by cinnamaldehyde in films were determined as the distance from color coordinates between sample films and control, following Equation (1)

(1) $$\Delta E=\sqrt{\Delta L^{2}+\Delta a^{*2}+\Delta b^{*2}}$$

Light transmission of sample and control films was measured by UV-vis spectroscopy following the procedure described earlier [31]. Additionally, the transparency parameter was calculated from the absorbance at the wavelength of 600 nm (A_600_) and the thickness of the film sample (t) in millimeters [32].

(2) $$\mathrm{Transparency}\;=\frac{A_{600}}{t}$$

All measurements were carried out in triplicate.

#### 2.8.2. Mechanical Tests

The mechanical characteristics of the dog bone specimens were evaluated in terms of elastic modulus (E); tensile stress (TS), and strain at break (ε_B_%) by submitting the specimens to stress–strain tests in a universal tensile test machine Testometric M350–20CT (Rochdale, UK), using the conditions described earlier [5]. The elastic modulus (E), defined as the relationship between the stress and the strain in the elastic zone of the materials, was calculated from the elastic region of the stress–strain curve employing the following equation:

(3) $$E=\frac{\sigma }{\varepsilon }$$

where σ is the stress in Pa and ε is the strain. To calculate the value of the tensile stress (TS), the following formula was employed:

(4) $$\mathrm{TS}=\frac{F_{\max}}{A}$$

where F_max_ is the maximum applied load in N and A is the original cross-sectional area (m^2^) of the dog bone specimens. Finally, the strain at break value, defined as the ratio between the increment in length after breakage and the initial length of the dog bone specimen, was calculated employing Equation (5).

(5) $$\varepsilon_{B}\left(\%\right)=100*\frac{\Delta L}{L}$$

where ΔL is the changed length and L is the initial length of the tested specimen. A minimum of five specimens were tested for each set of samples.

### 2.9. Antioxidant Properties

The antioxidant activity of the active films containing CIN was measured by the DPPH method. In its radical state, 2,2-diphenyl-1-pricylhydrazyl (DPPH) presents a maximum absorption at a wavelength of 517 nm but in the presence of an antioxidant or antiradical substance, the absorbance at the characteristic wavelength of 517 nm is decreased [33]. In this work, the procedure proposed by Valdés García et al., but with some modifications, was employed [34]. Briefly, pieces of film of about 1 cm^2^ were extracted with 4 mL of methanol at room temperature (RT) for 4 h. Then, 100 µL of the methanolic extract were mixed with 1.9 mL of freshly prepared methanolic solution of stable DPPH radicals (50 mg L^−1^). The solution was then shaken vigorously at RT and incubated in the dark for 60 min. All tests were performed in duplicate. The antioxidant activity was defined as the decrease in the absorbance of DPPH at 517 nm measured with a Jasco V-630 UV-vis spectrophotometer (Jasco Deutschland GmbH, Pfungstadt, Germany), and the radical scavenging activity (RSA) values were obtained following Equation (6):

(6) $$\mathrm{RSA}\;\left(\%\right)=\left(\frac{A_{c}-A_{s}}{A_{c}}\right)\times 100$$

where RSA (%) is the percentage of inhibition, A_c_ is the absorbance of the blank sample of DPPH methanolic solution and A_s_ is the absorbance of the tested sample at t = 60 min.

### 2.10. Antimicrobial Activity of EVOH-CIN Films

#### 2.10.1. Preparation of Fungal Cultures

*Penicillium expansum* was grown and maintained in Potato Dextrose Agar (PDA) plates for 7 days at 26 °C. Conidia suspension of *P. expansum* was obtained by pouring the surface of the PDA plates with sterile peptone water containing 0.05% (*v*/*v*) of Tween 80 and then dragging the surface with a digralsky spreader to favor detachment of conidia. Then, 1 mL sample of the fungal culture suspension was transferred to sterile tubes and were shaken to obtain a homogeneous suspension, which was carried out for several dilutions until obtaining 10^6^ spores/mL. The count of spores was conducted using improved Neubauer method (Bright-Line Hemacytometer, Hausser Scientific, Horsham, PA, USA).

#### 2.10.2. Antifungal Activity of Trans-Cinnamaldehyde

The in vitro antifungal activity of CIN was assessed in vapor phase by the disc diffusion method; the minimum inhibitory concentration (MIC) and the minimum fungicidal concentration (MFC) was determined as volume of aldehyde per plate against *P. expansum.* The minimum inhibitory amount was defined as the amount of cinnamaldehyde that causes 50% of inhibition with respect to the growth of the control after 7 days of incubation, whereas the fungicidal dose was the amount that completely inhibits the fungal growth after 10 days of incubation. The cinnamaldehyde doses per plate (µL/plate) were determined in Petri dish of PDA inoculated with 3 μL of 10^6^ spores/mL of conidia suspension in three equidistant points. Then tested volumes of cinnamaldehyde (1, 2, 5 and 10 μL) were added to 50 mm of sterile paper disk and placed on the lid of the plate, which were sealed with Parafilm to prevent leakage of compound. Controls were carried out without active compound. The plates were incubated at 26 °C for 7 days, after that time, paper disk was removed, and the plates were incubated for a further 3 days. The mean radial mycelial growth of *P. expansum* was evaluated by measuring the diameter of the fungal colony and was compared to control. All experiments were carried out in triplicate.

#### 2.10.3. Antifungal Activity of the Extruded Films

The antifungal activity of EVOH-CIN1, EVOH-CIN3 and EVOH-CIN5 films against *P. expansum* was evaluated by the micro-atmosphere method, subjecting the fungal culture to the atmosphere generated by the films. For that, 3 μL of 10^6^ spores/mL of conidia suspension were inoculated in three points of the sterile PDA plate, and a film sample of 0.3 g was placed on the inside of the lid, put on the plates and sealed with Parafilm. Then, they were incubated at 26 °C for 10 days, and the fungal growth was monitored by measuring the colony diameter after 3, 5, 7 and 10 days of incubation. The percentage of inhibition with respect to the control was calculated. After 7 days of incubation, the films were removed from the plates and were incubated for 3 more days to observe any fungicidal effect. Controls were conducted with films without cinnamaldehyde. All experiments were carried out in triplicate.

### 2.11. Statistical Analysis

Significance in the data differences of mechanical properties and antimicrobial activity values were statistically analyzed by one-way analyses of variance (ANOVA). For that aim, the Statgraphics Centurion XVII software version 17.2.04 was used. The identification of differences in the average values was evaluated by the Tukey test with a 95% confidence interval.

## 3. Results

### 3.1. Characterization of the EVOH-CIN15 Masterbatch

**Figure 1:**
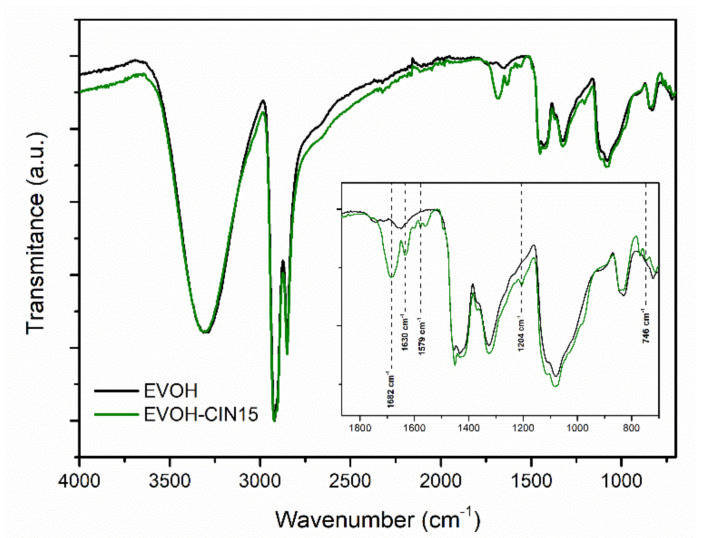
FTIR-ATR spectra of neat EVOH and EVOH-CIN15.

FTIR spectra of EVOH and EVOH incorporated with 15 wt.% of cinnamaldehyde by solution-extension-evaporation process are shown in Figure 1. The FTIR spectrum of pristine EVOH was characterized by several absorbance peaks typically found in EVOH copolymers such as those located at 2851 cm^−1^ (C–H), 2925 cm^−1^ (C–H) and 3340 cm^−1^ (O–H), which can be related to stretching vibrations, and at 1327 cm^−1^ (C–H) and 1450 cm^−1^ (C–H) due to bending vibrations [35,36]. The addition of cinnamaldehyde into the EVOH matrix led to the appearance of the characteristic bands of this bioactive compound: the peaks at 1682 and 1630 correspond to the stretching vibration of an aldehyde carbonyl C=O. A weak interaction may occur between CIN and the EVOH matrix based on the observation of the broad C=O signal of the cinnamaldehyde The band at 1579 cm^−1^ is associated with the aromatic ring C=C skeleton vibration of an aromatic compound, the band at 1204 cm^−1^ could be related to the C–H bending of the aldehyde group and the absorbance peak located at 746 cm^−1^ was attributed to the benzene rings=CH vibration absorptions [37], thus, confirming the successful incorporation of cinnamaldehyde into EVOH matrix. 

**Figure 2:**
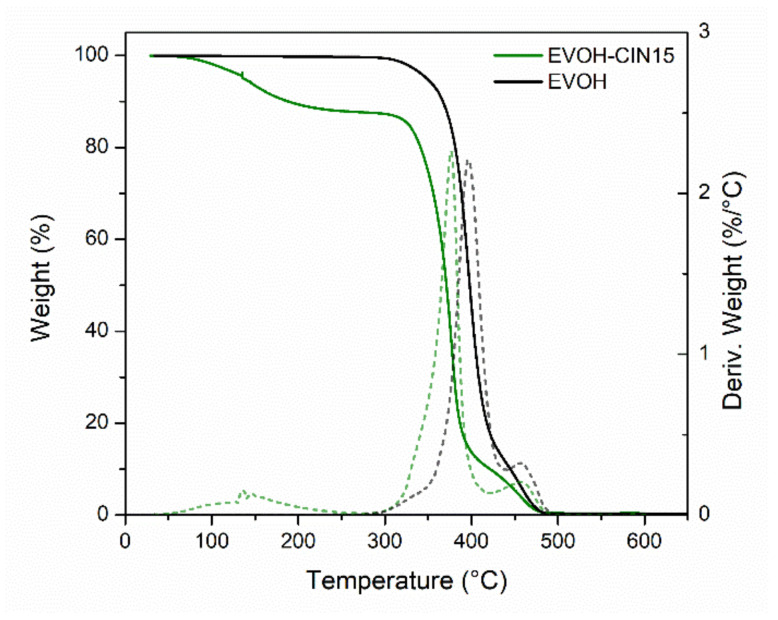
TGA and first derivative (DTG) curves of control EVOH and the masterbatch of EVOH incorporating 15 wt.% of cinnamaldehyde.

In order to calculate the real concentration of CIN present in the polymeric masterbatch, solid–liquid extractions with methanol were conducted at 40 °C. Then, liquid chromatography mass spectrometry was employed to determine the final concentration of the active ingredient, obtaining a value of 13.6 ± 0.2 g of cinnamaldehyde per 100 g of sample. This value was slightly below the 15% nominal concentration, likely owing to some loss associated to the volatilization of CIN or some decomposition caused during the preparation steps. Additionally, thermogravimetric analysis was employed as alternative method to estimate the final concentration of cinnamaldehyde incorporated in the polymeric masterbatch as well as to determine the different degradation processes occurring in the developed masterbatch. Figure 2 shows the TGA curves and their first derivates, which give the mass loss as a function of the temperature, of control EVOH and EVOH-CIN15 masterbatch. In EVOH-CIN15 it was possible to observe three main degradation processes. The first mass loss (not seen in neat EVOH) was observed between 90 and 250 °C, with a mass loss about 14%, and was attributed to the water evaporation and the volatilization of CIN. This value was in good accordance with the nominal incorporated amount of cinnamaldehyde in the EVOH matrix by the solution-extension-evaporation process. The second degradation stage was found between 250 and 430 °C, with a maximum temperature rate centered at 376 °C and was associated with the thermic elimination of side groups forming water, acetic acid, and acetaldehyde as by-products of the degradation reactions occurring in poly(vinyl) alcohol [38]. Finally, the third mass loss stage observed from 430 to 500 °C was attributed to the complete thermal degradation of ethylene vinyl alcohol copolymer backbones. 

### 3.2. Quantification of Cinnamaldehyde in EVOH Active Films after Melt-Processing

Four different formulations were developed by melt processing and coded as displayed in Table 1. EVOH-CIN15 masterbatch in powder form was blended with pristine EVOH pellets in different proportions, according to the masses in grams reported in Table 1, to obtain final weight ratios of cinnamaldehyde in relation to EVOH copolymer of 1, 3 and 5 wt.%. The high heat reached in the melt processing with temperature values above 170 °C, together with the high volatility of cinnamaldehyde can result in significant losses of the final amount of CIN in the extruded films, therefore, liquid chromatography mass spectrometry (LC-MS) was employed to determine the concentration of CIN present in the active EVOH-based samples after melt-processing.

The results showed that the loss of cinnamaldehyde after melt-processing was around a 40%, without regard to the starting quantity of CIN present in the formulations. This result was attributed to some losses of cinnamaldehyde during the preparation of the polymeric masterbatch and, on the other hand, to the vaporization of cinnamaldehyde by the high temperatures and shear stresses during melt processing. However, previous works dealing with the use of similar volatile compounds such as thymol and R-(-)-carvone in extrusion processing reported that the remaining amount of the volatile agents in the films were decreased to lower than 50% of the starting loading concentrations [39]. Other study reports a thymol loss of 70% when this additive was extruded with polyethylene (PE), attributed to the effect of temperature and to the low chemical affinity between the PE matrix and the natural compound [40]. In this line, the hybrid technique employed in this work, consisting of a two-step protocol involving the prior incorporation of cinnamaldehyde in the EVOH-based masterbatch by solvent casting followed by the melt extrusion step, can potentially minimize losses of this volatile compound during the melt extrusion process.

### 3.3. Structural and Morphological Properties of the Films

#### 3.3.1. FTIR Analysis

**Figure 3:**
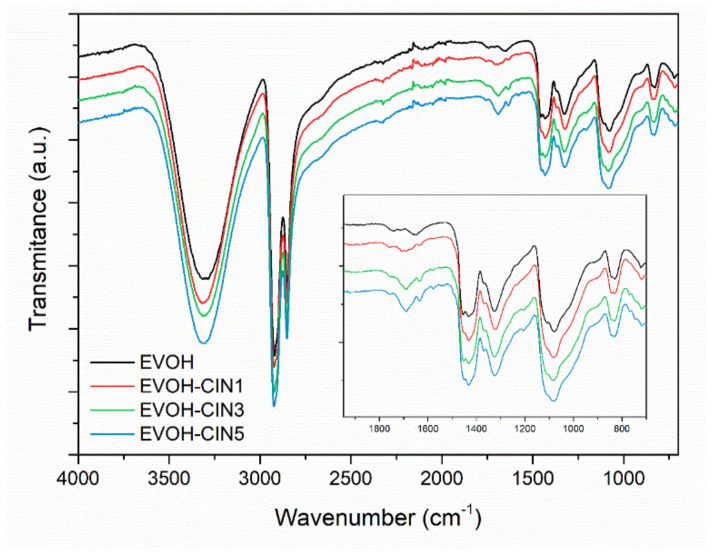
FTIR-ATR spectra of EVOH and EVOH films incorporated with cinnamaldehyde.

FTIR spectrum of melt-processed control EVOH and spectra of EVOH-cinnamaldehyde active films are displayed in Figure 3. Spectra were vertically translated for a proper visualization and the insert includes the spectra of the samples in the region of interest. On the one hand, EVOH copolymer showed the characteristic bands described in Section 3.1. On the other hand, the addition of the EVOH-cinnamaldehyde masterbatch resulted in the appearance of the aforementioned characteristic peaks of cinnamaldehyde at 1682 and 1630 cm^−1^ (νC=O), 1579 cm^−1^ (νC=C), and 746 cm^−1^ (benzene rings=CH vibration), indicating that cinnamaldehyde remains after the extrusion process. In addition, these bands increase in intensity as the content of EVOH-CIN15 masterbatch augments in each formulation.

#### 3.3.2. SEM Analysis

**Figure 4:**
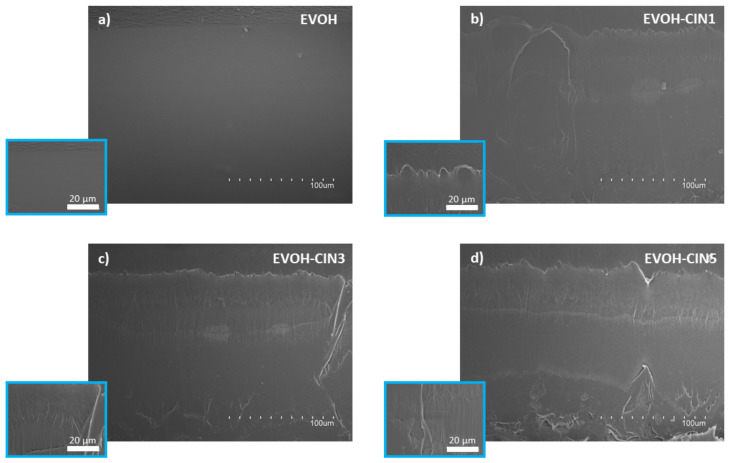
Film morphology: SEM images of cryofracture surface of (**a**) EVOH, (**b**) EVOH-CIN1, (**c**) EVOH-CIN3 and, (**d**) EVOH-CIN5 at magnifications of 500× and 2000× (inserts).

The SEM microstructure images of the cross sections of the EVOH film and those incorporated with different amounts of cinnamaldehyde are represented in Figure 4. A smooth, homogeneous, and clean broken surface was observed for neat EVOH film (Figure 4a). Meanwhile, a single and homogenous phase, with some ductile fracture patterns, was observed in the cross sections of EVOH containing different amounts of cinnamaldehyde, indicating that cinnamaldehyde did not significantly influence EVOH morphological structure (Figure 4b–d). The plastic regions observed in the films where cinnamaldehyde was present, can be associated with a plasticizing effect of the antimicrobial compound, since the introduction of low molecular weight additives such as cinnamaldehyde, could potentially reduce the molecular interactions occurring between atoms along the polymer chain, favoring the solubilization of the additive into the EVOH matrix, thus, leading to a more plastic behavior [41].

### 3.4. Film Thermal Properties

Figure S1 displays the DSC curves of extruded films during the heating and cooling cycles while Table 2 reports the main thermal parameters extracted from these curves. Neat EVOH has a glass transition temperature (T_g_) located at 55 °C, according to manufacturer’s value. During the first heating scan it was not possible to well-define its T_g_. The incorporation of different amounts of cinnamaldehyde resulted in a decrease in the T_g_ values, of up to 14 °C in EVOH-CIN1 formulation. This decrease in the T_g_ was associated with the ability of cinnamaldehyde to act as a plasticizer, increasing the mobility of polymer chains, caused by an increment in the free volume between them [42]. Prior to the melting process, no endothermic peak associated with a potential loss or degradation of cinnamaldehyde during the first heating was observed, probably because of the low amount of the compound in the active formulations. The melting temperature reported for neat EVOH, located at 166 °C, was in good accordance with the manufacturer’s value for EVOH pellets. This may be related to the preparation method employed for the film formation, since extrusion-processing favors higher melting temperatures and melting enthalpy as compared to the solvent-casting fabrication process, since a more efficient crystallinity structure is obtained [43]. The presence of cinnamaldehyde in EVOH-based films resulted in a clear reduction in both the melting temperature and enthalpy, which decreased when increasing the cinnamaldehyde content. This effect had an influence on the crystallinity degree values, that decreased by increasing cinnamaldehyde amount. The lower energy required to melt the crystalline structures suggests that some structural modifications have taken place that affect the crystallization process. Regarding the crystallization temperature (T_c_) during the cooling scan, an evident decrease in the T_c_ value was observed in EVOH-CIN5 formulation with respect to neat EVOH, indicating that cinnamaldehyde may favor the crystallization of EVOH at lower temperatures.

During the second heating scan, it was possible to define the glass transition of neat EVOH, centered at 54 °C. As shown during the first heating cycle, the presence of cinnamaldehyde resulted in a decrease of T_g_ in the active formulations, but higher values of glass transition temperatures were reported with respect to the first heating scan, suggesting that a loss of cinnamaldehyde may occur after the first heating cycle. On the other hand, no significant changes were observed during the second heating cycle in both the melting temperatures and the calculated degree of crystallinity of the developed formulations with respect to the first heating cycle.

**Figure 5:**
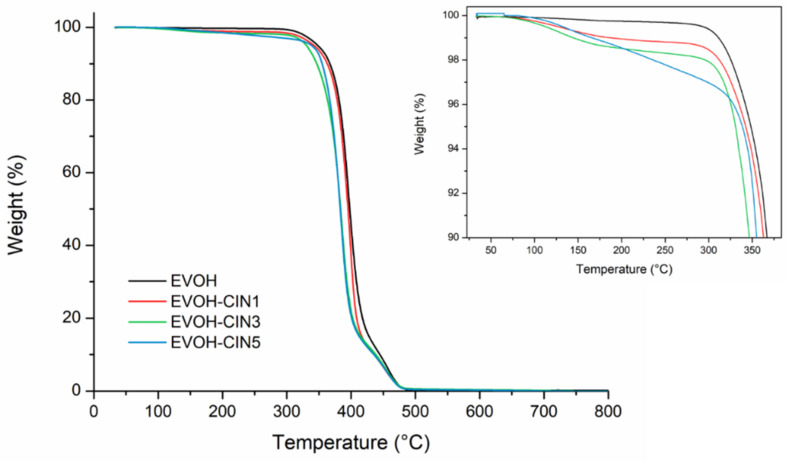
Thermogravimetric curves of control EVOH and EVOH incorporated with CIN.

To evaluate the influence of the incorporation of cinnamaldehyde over both, the EVOH matrix thermal stability and over its thermal degradation process, TGA was carried out under inert atmosphere. Figure 5 displays the mass loss vs. temperature thermogravimetric curves for EVOH and EVOH incorporated with cinnamaldehyde. Table 3 reports the initial degradation temperature and the temperature at maximum weight loss rates.

The TG curves showed that the degradation process for all films occurred in two main degradation steps. The insert shows a small weight loss in the TG curve from 125 to 300 °C, preceding the main degradation stages of the EVOH copolymer matrix, which was ascribed with the cinnamaldehyde vaporization and to the presence of adsorbed water. The weight loss percentage of each formulation in that temperature range was consistent with the theoretical amount of cinnamaldehyde in the film and with the results obtained from the LC-MS analysis. This phenomenon was responsible for the decrease in the onset temperature value (T_5%_) of EVOH films containing cinnamaldehyde, compared to the EVOH control film. Regarding the temperatures at maximum weight loss rates, the first main degradation process in the EVOH control film was located at 397 °C and was ascribed to thermic removal of side chain groups and degradation of low molecular weight by-products occurring during the thermal degradation of PVOH, as described in Section 3.1 [38,44]. Cinnamaldehyde presence shifted the T_maxI_ to lower temperatures, indicating that some interaction may occur between vinyl alcohol component of EVOH copolymer and cinnamaldehyde. In addition, it could be associated with some losses of remaining amount of cinnamaldehyde. The complete thermal degradation of ethylene vinyl alcohol copolymer backbone took place in the second stage at higher temperatures (457 °C). The incorporation of cinnamaldehyde into EVOH films resulted in no significant differences in the maximum peak temperature of the second degradation stage.

### 3.5. Functional Properties of the Film

#### 3.5.1. Thickness and Optical Properties

As expected, control EVOH resulted in colorless and transparent films. After cinnamaldehyde addition, the chromaticity of EVOH films developed by extrusion processing slightly changed to yellow tones. The possibility of seeing through the film is one of the most important features influencing the decision of consumers in the food packaging sector [45]. In this line, colorimetric analysis and light transmission analysis in the visible light region were performed and the results are displayed in Table 4 and Figure S2, respectively. All films were characterized by a high lightness value (*L* > 95), confirming their high transparency, as already observed by visual evaluation. EVOH films presented a* and b* values of −0.2 and 0.5, respectively. Positive values were registered in melt processed EVOH incorporated with different amounts of cinnamaldehyde for b* parameter, confirming a deviation toward yellow chromaticity. Moreover, small but negative values in a* coordinate showed a slight trend to green in EVOH-CIN films with respect to neat EVOH. These variations had as a consequence total color difference values (Δ*E*) of EVOH films incorporated with cinnamaldehyde ranging between 3.9 and 10.7, indicating that the color changes provoked by the addition of cinnamaldehyde are noticeable by an unexperienced observer. In addition, the yellow index was also calculated and used to judge the yellowness of the developed films. It could be observed that yellow index (YI) values increased as the content of cinnamaldehyde in each formulation augmented, in good agreement with visual observations.

Regarding the UV-vis light measurements, EVOH film presented a great transparency along the visible region of the spectra between 400 and 800 nm (see Figure S2). The presence of cinnamaldehyde in EVOH based films resulted in a slight decrease in the transparency values as reported in Table 4. Interestingly, cinnamaldehyde produced a blocking effect in the UV region of the spectra (250–400). This effect has been already reported in other works utilizing different natural compounds [46,47]. The better UV-vis light barrier properties of EVOH films incorporated with cinnamaldehyde can potentially minimize the accelerated decomposition of some photosensitive food produce since this kind of light may cause lipid oxidation in food [48].

#### 3.5.2. Mechanical Properties

The mechanical performance of packaging films intended to protect packaged products is of great importance since the mechanical properties show the capacity of a certain material to preserve its integrity under loading during the stages of processing, handling, and storage. For this purpose, the effect of the incorporation of cinnamaldehyde in the tensile properties of EVOH copolymer was studied. The results of the mechanical properties of dog bone specimens subjected to tensile tests for all developed formulations are reported in Table 5. Control EVOH presented an elastic modulus of 3.2 GPa, a tensile stress of 62 MPa and an elongation at break value of 33%. The addition of cinnamaldehyde mainly maintained the Young’s modulus, slightly increased the tensile strength and at the same time a remarkable plasticizing effect was observed in the formulations containing cinnamaldehyde, specifically in EVOH samples containing 3 and 5 wt.% of CIN. This result was in full agreement with the decrease in their glass transition temperature values, related to the increment in the mobility of EVOH polymer chains caused by the plasticizing effect of cinnamaldehyde, as described in the DSC analysis. The plasticizing effect of CIN has been previously reported in poly(lactic acid)/poly(trimethylene carbonate) films and has been attributed to the phase slipping provoked by the low molecular weight compound incorporated into the polymeric matrix [42].

### 3.6. Antioxidant Properties

The inhibition of lipid oxidation by enriching packaging films with antioxidant substances is a very promising way to extend the shelf life of food produce. Cinnamaldehyde has been mainly utilized to prepare active packaging films with antimicrobial activity, but this bioactive compound has also demonstrated a moderate antioxidant activity [19,49,50]. In this line, the DPPH assay was used to evaluate the ability of CIN extracted from the active films to act as free radical scavenger or hydrogen donor in order to determine its antioxidant activity. As expected, control EVOH did not present any antioxidant activity. On the other hand, bioactive films incorporated with 1, 3 and 5 wt.% of cinnamaldehyde showed radical scavenging activity (%) values of 24, 30, and 32%, respectively. After performing a second and subsequent extraction step no radical scavenging activity was observed in any formulation, indicating that cinnamaldehyde was totally released from the polymer matrix into methanol during the first extraction step. The obtained results confirmed that the addition and the presence of low concentrations of cinnamaldehyde in the active materials conferred some antioxidant activity to the EVOH films with respect to pristine EVOH. DPPH test shows the radical scavenging properties of molecules capable of either transferring an electron or donating hydrogen. Although CIN has considerable metal ion chelating ability [51], it presents moderate antioxidant activity as the above results show. In this line, radical scavenging activity of CIN has been reported to be much lower than that of its derivatives cinnamyl alcohol and cinnamic acid [49] or that of molecules with hydroxyl substituents on the aromatic ring in phenolic compounds such as eugenol [51].

### 3.7. Antimicrobial Activity

#### 3.7.1. Antifungal Activity of Cinnamaldehyde

The antifungal effectiveness of cinnamaldehyde in vapor phase against *P. expansum* was evaluated by determining the MIC and MFC, which were found to be 2 and 5 μL/plate, respectively. These results show that CIN exerted a great activity against P. expansum and only a small amount of the volatile was necessary to produce a great inhibition of the fungus. Although the mechanism of action of CIN is not clear, previous studies suggest that the lipophilic character of the volatile allows its interaction with the cell membrane, interfering in permeability and structural integrity of the cell, which affects to normal fungal growth [52,53]. Moreover, other authors reported the ability of α,β-unsaturated aldehydes to react with biologically essential nucleophilic groups as sulfhydryl groups of amino acids or DNA to form adducts [54]. The potential of cinnamaldehyde as antifungal compound has been previously reported showing a great activity against several strains of *Penicillium* sp. when was applied in vapor phase [55,56,57]. The present results show that cinnamaldehyde is a good candidate to be used as an antimicrobial agent in the development of active films for food packaging.

#### 3.7.2. Antifungal Activity of Extruded Films

**Figure 6:**
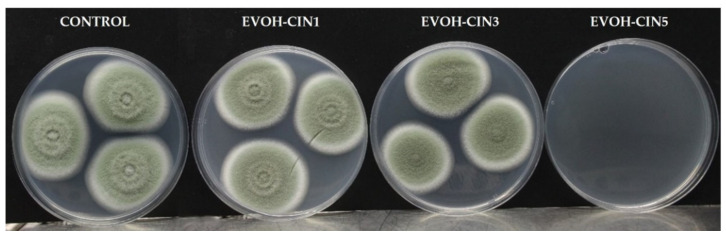
Antifungal effect of extruded EVOH films after 7 days of incubation at 26 °C.

Antifungal activity of EVOH-CIN films incorporated with different amounts of CIN is displayed in Table 6 whereas pictures of fungal growth are shown in Figure 6. According to the results obtained, EVOH-CIN5 completely inhibited the growth of *P. expansum* exerting a fungicidal effect since no fungal growth was observed during the days following the removal of the film from the inside of the lid at day seven. Regarding EVOH-CN1 films, no fungal inhibition was observed whereas the morphology of the fungal colony was affected in presence of EVOH-CIN3 since a reduction in mycelial density was observed.

## 4. Conclusions

A hybrid method for the preparation of bioactive EVOH films incorporated with cinnamaldehyde was employed and successfully applied to develop antifungal and antioxidant EVOH-based films by melt processing. Prior to the extrusion step, the corrected preparation of an EVOH-CIN masterbatch was confirmed by FTIR and thermogravimetric analysis. Then, different EVOH-CIN extruded films were obtained, and a complete characterization was carried out. The results described in this work show the feasibility of the employed method to obtain bioactive films by industrial processing extrusion technique with enhanced functionalities thanks to their antioxidant, UV barrier and antifungal properties, which are of great interest in food packaging applications.

## Figures and Tables

**Table 1:** EVOH-based formulations and final concentration of cinnamaldehyde determined by liquid chromatography mass spectrometry (LC-MS) in the active films after melt processing (wt.%). Mean ± SD (n = 3).

Reference	Neat EVOH (g)	EVOH-CIN15 (g)	Cinnamaldehyde (wt.%)
EVOH	14	0	Not detected
EVOH-CIN1	13.07	0.93	0.58 ± 0.02
EVOH-CIN3	11.20	2.80	1.72 ± 0.03
EVOH-CIN5	9.30	4.70	3.12 ± 0.08

**Table 2:** Thermal properties of control EVOH and EVOH-CIN formulations obtained from the DSC curves during the heating and cooling cycles.

	First Heating Scan				Cooling Scan			Second Heating Scan			
Formulation	T_g_ (°C)	T_m_ (°C)	∆H_m_ (J/g)	χ_c_ (%)	T_g_ (°C)	T_c_ (°C)	∆H_c_ (J/g)	T_g_ (°C)	T_m_ (°C)	∆H_m_ (J/g)	χ_c_ (%)
EVOH	-	166	79.7	37	58	142	71.2	54	166	83.5	38
EVOH-CIN1	41	165	69.6	32	46	142	63.0	49	163	74.0	34
EVOH-CIN3	44	163	62.9	30	46	141	63.3	48	162	67.1	32
EVOH-CIN5	43	157	60.9	29	46	134	53.3	48	157	62.2	30

**Table 3:** Maximum degradation temperatures of EVOH and EVOH-CIN films.

Formulation	ΔW_30–300 °C_ (%)	T_5%_ (°C)	T_maxI_ (°C)	T_maxII_ (°C)
EVOH	0.2	348	397	457
EVOH-CIN1	1.3	342	398	454
EVOH-CIN3	2.1	330	386	453
EVOH-CIN5	3.0	338	384	454

**Table 4:** Thickness, color parameters obtained from CIELab space and YI.

Formulation	Thickness (µm)	*L*	a*	b*	Δ*E*	YI	Transparency (A_600_/t)
EVOH	181 ± 3	99.1 ± 0.2	−0.2 ± 0.1	0.5 ± 0.1	-	0.6 ± 0.1	509 ± 4
EVOH-CIN1	193 ± 9	98.1 ± 0.1	−1.1 ± 0.1	4.2 ± 0.2	3.9 ± 0.3	6.1 ± 0.3	464 ± 11
EVOH-CIN3	202 ± 5	98.4 ± 0.0	−1.6 ± 0.1	6.0 ± 0.5	5.7 ± 0.5	8.6 ± 0.7	441± 2
EVOH-CIN5	204 ± 6	97.7 ± 0.3	−2.8 ± 0.6	10.8 ± 2.7	10.7 ± 2.8	15.2 ± 3.7	404 ± 8

**Table 5:** Mechanical properties (mean ± SD; n = 5).

Formulation	Young’s Modulus (GPa)	Tensile Strength (MPa)	Elongation at Break (%)
EVOH	3.2 ± 0.0 ^a^	62 ± 1 ^a^	33 ± 3 ^a^
EVOH-CIN1	3.3 ± 0.1 ^a,b^	65 ± 4 ^b^	31 ± 4 ^a^
EVOH-CIN3	3.3 ± 0.1 ^b^	66 ± 1 ^b^	41 ± 14 ^b^
EVOH-CIN5	3.3 ± 0.1 ^b^	69 ± 2 ^c^	67 ± 4 ^c^

^a–c^ Different letters in the same column indicate a statistically significant difference (*p* < 0.05).

**Table 6:** Antifungal effectiveness of EVOH-CIN films with 1, 3 and 5 wt.% of cinnamaldehyde against *Penicillium expansum* for 10 days at 26 °C.

Inhibition of *P. expansum* (%)				
Time (days)	3	5	7	10
EVOH-CIN1	9.1 ± 3.9 ^a^	3.5 ± 2.1 ^a^	2.8 ± 1.6 ^a^	3.6 ± 2.7 ^a^
EVOH-CIN3	25.2 ± 8.2 ^b^	15.1 ± 4.7 ^b^	7.1 ± 2.9 ^b^	4.7 ± 3.6 ^a^
EVOH-CIN5	100 ^c^	100 ^c^	100 ^c^	100 ^b^

^a–c^ Different letters in the same column indicate a statistically significant difference (*p* ≤ 0.05).

## Supplementary Materials

The following are available online at https://www.mdpi.com/article/10.3390/foods10071591/s1, Figure S1: First heating DSC curves (a), cooling DSC curves (b), and second heating DSC curves (c) of EVOH and EVOH-CIN films; Figure S2: UV-vis light transmission of films in the range of 250–800 nm.
