# Supplementary material for: Hot-Melt-Extruded Active Films Prepared from EVOH/Trans-Cinnamaldehyde Blends Intended for Food Packaging Applications
Source: Foods. 2021 Jul 8;10(7):1591. doi: 10.3390/foods10071591 (PMC8304191; doi:10.3390/foods10071591)
Supplement: Supplementary file 1 [file foods-10-01591-s001.zip › foods-1259945-supplementary.pdf]

SUPPORTING INFORMATION

Hot-melt-extruded Active Films Prepared from EVOH/trans-cinnamaldehyde Blends Intended for Food Packaging Applications

Alejandro Aragón-Gutiérrez [1,*], Raquel Heras-Mozos [2], Miriam Gallur [1], Daniel López [3], Rafael Gavara [2] and Pilar Hernández-Muñoz [2,*]

[1] Grupo de Tecnología de Envases y Embalajes, Instituto Tecnológico del Embalaje, Transporte y Logística, ITENE, Unidad Asociada al CSIC, calle de Albert Einstein 1, 46980 Paterna, Valencia, Spain; miriam.gallur@itene.com (M.G.)

[2] Instituto de Agroquímica y Tecnología de Alimentos, IATA-CSIC, calle del Catedrático Agustín Escardino Benlloch 7, 46980 Paterna, Valencia, Spain; r.heras@iata.csic.es (R.H.-M.) rgavara@iata.csic.es (R.G.)

[3] Instituto de Ciencia y Tecnología de Polímeros, ICTP-CSIC, calle Juan de la Cierva 3, 28006 Madrid, Spain; daniel.l.g@csic.es (D.L.)

* Correspondence: alejandro.aragon@itene.com (A.A.-G.); phernan@iata.csic.es (P.H.-M.); Tel.: +34-961 820 000 (A.A.-G.); +34-963 900 022 (P.H.-M.)

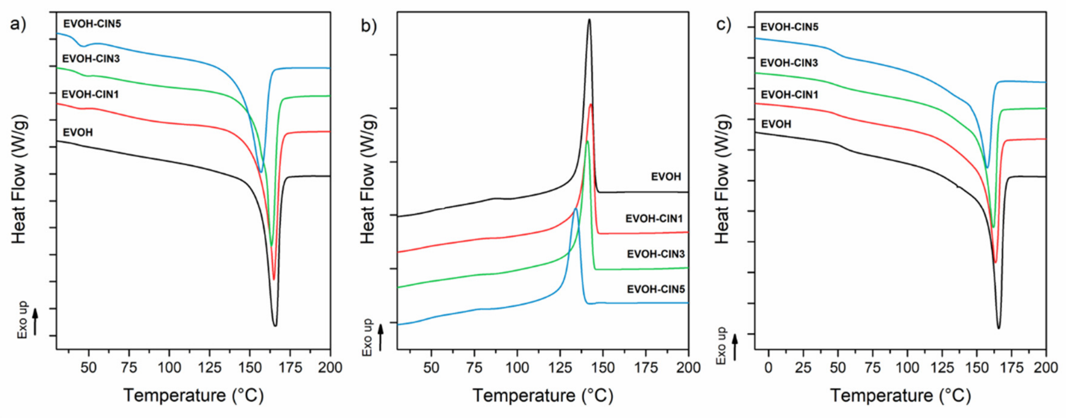

Figure S1. First heating DSC curves (a), cooling DSC curves (b), and second heating DSC curves (c) of EVOH and EVOH-CIN films

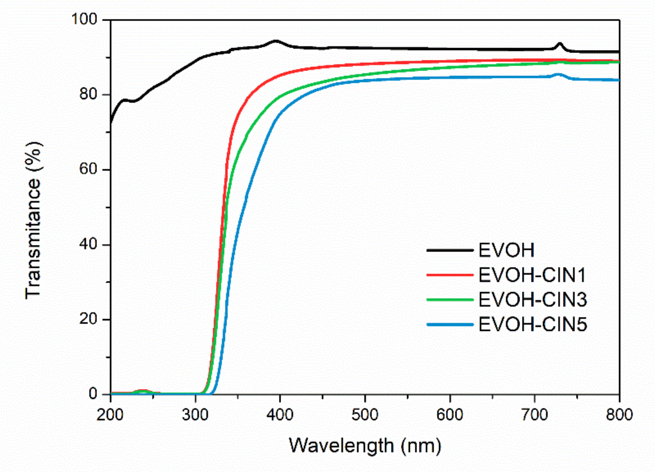

Figure S2. UV-vis light transmission of films in the range of 250-800 nm

****
